# Medical and Physical Intelligence

**Published:** 1806-03

**Authors:** 


### MEDICAL AND PHYSICAL INTELLIGENCE.

The Galvanic Society at Paris have attempted, but without fuccefs, to follow M. Pacchiani in the decompofition of the muriatic acid. After a feries of accurate experiments, they declared it as their opinion, that the Profeffor was deceived as to the nature of the acid which it was announced that he obtained, and that it was probably produced by fome animal or vegetable fubftance employed in the apparatus: they add alfo that they do not think it poffible to effect any thing by the action of the Galvanic pile, but a decompofition of the water ufed in the experiment.

M. Pully, a French chemift, has lately employed himfelf in analyfing Dr. James's powder; and as the refult of many experiments, he fays of nineteen parts of James's powder, there are of

| | *Parts.* |
|---|---|
| Oxyd of antimony — — — | 7 |
| Phofphat of lime — — — | 4 |
| Sulphat of potafh — — — | $4\frac{1}{2}$ |
| Free potafh, containing oxyd of antimony | $3\frac{1}{2}$ |
| | 19 |

To recompofe this powder, he takes

| | |
|---|---|
| Sulphat of antimony — — — | 2 |
| Calcined phofphat of lime — — | $1\frac{1}{2}$ |
| Nitrat of potafh — — — | 4 |
| | $7\frac{1}{2}$ |

Thefe fubftances are to be pulverized, mixed, and triturated; they are then to be put into a crucible, which is to be clofed, and ftrongly heated. During this operation, the oxygen of the nitric acid, acting upon the fulphur of the antimony, converts it into fulphuric acid, which unites with a portion of the potafh, and forms fulphat of potafh; the reft of the free potafh retains antimony oxydated at the minimum. The white powder which remains in the crucible is the fame as that known by the name of Dr. James's powder.

A Summary of Mr. Brande's experiments on guaiacum was read to the Royal Society, at one of its meetings in Jannary; from which it appears, that in 100 grains of that fubftance there were $22\frac{1}{2}$ of water and oil; of empyrheumatic oil about 30; carbon 30; lime 9; carbonated hydrogen gas about 8. On the fame and on a fubfequent evening was read a letter from F. A. Knight, Efq. to the Prefident, on the defcent of the roots, and the elongation of the germs of plants: in this the author has endeavoured to determine whether the defcent of the roots of plants was the effect of an inherent principle, or the confequences of mechanical gravitation; and and he determines that gravitation is the ſole cauſe of the deſcent of roots and aſcent of germs; and that both diverge in all directions, when under the influence of equal preſſure.

---

Mr. Hatchett has laid before the Royal Society a third communication on artificial tanning: he finds that all gums, reſins, and balſams, afford this ſubſtance on being treated with nitric acid. They all yield at one operation a certain quantity of this matter; but if the proceſs be continued too long the product is deſtroyed.

---

The following reſults have been given from experiments made on the torpedo by Meſſrs. Humboldt and Lusac: 1. Though the ſtrength of the torpedo is far inferior to that of the gymnotus, it is equally capable of cauſing painful ſenſations. 2. The gymnotus gives the moſt violent ſhocks, without any exterior movement of the eyes, the head, or the fins; but the torpedo ſuffered a convulſive movement of the pectoral fins each time it gave a ſhock. 3. The powers of theſe fiſh cannot be excited at pleaſure; they muſt be irritated before they will give a ſhock; of courſe the action depends on the will of the animal. 4. The ſhock is equally felt on touching with one finger a ſingle ſurface of the electric organs, as on applying the two hands to the two ſurfaces at once. 5. When an inſulated perſon touches the torpedo with a ſingle finger, the contact muſt be immediate, as no ſhock will be felt if a conducting body, of metal for example, be interpoſed between the finger and the organ of the fiſh; but if both hands are uſed, one touching the fiſh, and the other the metal, a ſevere ſhock will be felt. 6. The moſt ſenſible electrometer manifeſts no electrical tenſion in the organs of the torpedo, in whatever way it is applied. The leaſt injury on the brain of the torpedo deſtroys its electric powers.

---

Dr. Struve, a ſkilful phyſician of Gorlitz, has invented a machine, the object of which is to apply galvaniſm to the purpoſe of diſtinguiſhing real from apparent death.

---

M. Klaproth, a ſhort time before his death, diſcovered that the ſolution of the metallic oxyds in alkalis are as eaſily precipitated in their metallic ſtate, by the other metals ſoluble in the ſame alkalis as the acid ſolutions of theſe metals are by phoſphorus. He had made a very ingenious application of this proceſs to the analyſis of tin ores. In the operation, tungſtein is ſeparated from tungſtate of ammonia, by the addition of zinc, in the form of black flakes.

---

A Correspondent, in a letter, dated Colchester, Jan. 20, 1806, writes as follows: — "Having served in his Majesty's forces for many years as a Surgeon, and supposing I had laid a good foundation to qualify myself for a Physician, I went to Cambridge with a view of keeping Terms, and attending the Lectures which that University prescribes, but was informed that my practice in the army was an insuperable impediment, as a decree was made last year year, 'That no one engaged in any trade or profession could proceed to the degree of Bachelor of Physic. This surprized me much, especially when I was told that no Medical Lectures were given by the Professor of Physic; nor was my surprize diminished, when I found that in the same month the Admiralty had made a regulation, 'that no one should act as a Physician in their department, who had not practised as a Surgeon at least five years.' Perhaps some of your correspondents may explain what to me seems paradoxical, that the practice which recommends a medical man to the world, should disqualify him as a Physician in the eyes of a University. It follows therefore, that only *Scotch* Physicians, or those who have taken no degree at all, can be employed in the navy."

---

Dr. ARNOLD, of Leicester, whose valuable Work *on Insanity* has long been out of print, and been sold at high prices, is preparing a new and enlarged Edition for immediate publication.

---

Dr. ADAMS has gone to prefs with a new edition of "Morbid Poifons." Though the principles contained in this new edition are the fame as in the laft, the conftruction of the work is different. The controverfial part is omitted, excepting as far as it may illuftrate Mr. Hunter's opinions, which are reduced to practical rules. Yaws, Sivvens, and Elephantiasis, are defcribed from the Author's clinical obfervations; and the anomalous poifons which have been confounded with the venereal, are arranged according to diftinct characters, their illuftrations being greatly enriched by publications fince the former edition, and by the Author's own obfervations.

---

Dr. PINCKARD's Notes, containing an Account of the Expedition to the Weft Indies, under the command of the late General Sir Ralph Abercromby and the late Admiral Sir Hugh C. Chriftian, with Obfervations refpecting the Difeafes of the Weft Indies, and particularly regarding the "Seafoning" or "Yellow" Fever of hot Climates, may be fpeedily expected.

